# Beyond Risk Reduction: Vigilant Trust in Artificial Intelligence Based on Evidence from China

**DOI:** 10.3390/bs16010095

**Published:** 2026-01-09

**Authors:** Wuyao Ding, Yun Wu, Junxiu Wang

**Affiliations:** 1 School of Mental Health, Wenzhou Medical University, Wenzhou 325035, China; dingwy@wmu.edu.cn; 2 School of Media and Communication, Shanghai Jiao Tong University, Shanghai 200240, China; 3 Chinese Academy of Social Sciences, Beijing 100732, China; yunwu@cass.org.cn

**Keywords:** trust, vigilance, AI acceptance, perceived risks, perceived benefits

## Abstract

Public trust in artificial intelligence (AI) is often assumed to promote acceptance by reducing perceived risks. Using a nationally representative survey of 10,294 Chinese adults, this study challenges that assumption and introduces the concept of vigilant trust. We argue that trust in AI does not necessarily diminish risk awareness but can coexist with, and even intensify, attention to potential harms. By examining four dimensions of trust—trusting stance, competence, benevolence, and integrity—we find that all of them consistently enhance perceived benefits, which emerge as the strongest predictor of AI acceptance. However, trust shows differentiated relationships with perceived risks: benevolence reduces risk perception, whereas trusting stance is associated with higher perceptions of both benefits and risks. Perceived risks do not uniformly deter acceptance and, in some contexts, are positively associated with willingness to adopt AI. By moving beyond the conventional view of trust as a risk-reduction mechanism, this study conceptualizes vigilant trust as a mode of engagement in which openness to AI is accompanied by sustained awareness of uncertainty. The findings offer a more nuanced understanding of public acceptance of AI and its implications for governance and communication.

## 1. Introduction

Artificial intelligence (AI) technologies are rapidly permeating everyday life—from generative models and autonomous vehicles to predictive systems used in finance, healthcare, and public administration. While these systems hold considerable promise, they also raise concerns related to privacy, fairness, security, and accountability (Al-kfairy et al., 2024). Recent global surveys consistently show that citizens recognize AI’s potential while simultaneously expressing anxiety, uncertainty, and fear (Brauner et al., 2025; Kouloukoui et al., 2025; Okoidigun & Emagbetere, 2025; Said et al., 2023). Public evaluations of AI, therefore, tend to be ambivalent rather than uniformly positive or negative, reflecting a persistent tension between optimism and caution. Understanding how such mixed perceptions translate into acceptance has become an increasingly central question for scholars and policymakers (Chow et al., 2023; Kelly et al., 2023).

Among the prevailing approaches to technology adoption, the Technology Acceptance Model (TAM; Davis, 1989) and the Unified Theory of Acceptance and Use of Technology (UTAUT; Venkatesh et al., 2003) remain foundational frameworks for explaining the uptake of conventional, well-structured technologies (Kelly et al., 2023). TAM emphasizes perceived usefulness and ease of use (Davis, 1989), while UTAUT incorporates performance expectancy, social influence, effort expectancy, and facilitating conditions (Venkatesh et al., 2003). While demonstrating strong predictive power for non-intelligent technologies, these models were not originally developed to account for uncertainty, opacity, and potential harm, limiting their ability to capture the risk-oriented evaluations that increasingly define public responses to AI (Gursoy et al., 2019; Sohn & Kwon, 2020).

Reflecting this shift, recent research has extended acceptance models by explicitly incorporating perceived risks alongside perceived benefits, acknowledging that individuals often weigh opportunities and potential harms simultaneously when forming attitudes toward AI (Said et al., 2023; Schwesig et al., 2023). Within these expanded frameworks, trust has emerged as a pivotal antecedent shaping how people interpret AI’s advantages and uncertainties (Kelly et al., 2023; Kenesei et al., 2022; Kerstan et al., 2024; Zhang et al., 2021). However, most trust-based extensions continue to conceptualize trust primarily as a risk-reducing mechanism, assuming that greater trust dampens perceptions of harm and thereby facilitates acceptance (Kim et al., 2008; Habbal et al., 2024).

Accumulating evidence suggests that this assumption does not always hold. Research on critical trust, for example, shows that individuals may sustain trust in institutions or technologies while simultaneously engaging in scrutiny and oversight, rather than suspending judgment altogether (Porta, 2013; Walls et al., 2004). While this work highlights that trust and critique can coexist at a normative or attitudinal level, it provides limited insight into the cognitive mechanisms through which such coexistence is enacted. Epistemic vigilance offers a cognitively grounded perspective on this issue. Originating in cognitive and evolutionary psychology, epistemic vigilance refers to the mechanisms through which individuals regulate the acceptance of communicated information by monitoring the reliability of sources and content (Mercier, 2020; Sperber et al., 2010). Importantly, epistemic vigilance is neither equivalent to philosophical skepticism, which assumes that we can never be certain because we cannot rule out all potential errors, leading to the rejection of knowledge claims altogether (Stone, 2000). Rather, it conceptualizes trust and vigilance as mutually constitutive processes, allowing openness to information to coexist with ongoing evaluative scrutiny. This perspective helps explain how individuals may simultaneously recognize both the potential benefits and risks of AI, and why trust does not necessarily suppress sensitivity to uncertainty. Building on this logic, the present study conceptualizes vigilant trust as a mode of trust in which openness and cognitive scrutiny co-occur within a single evaluative process.

Beyond how trust shapes benefit and risk perceptions, another unresolved issue concerns the functional role of perceived risk in technology acceptance. Although perceived risks are now routinely incorporated into acceptance models (e.g., Bedué & Fritzsche, 2022), their effects remain theoretically ambiguous. While some studies report the expected negative association between risk and acceptance (Lee, 2009), others find null or even positive relationships (S. Wang et al., 2019). These inconsistencies suggest that perceived risk does not operate solely as a deterrent but may play more complex roles in sociotechnical contexts where AI systems are highly capable, widely promoted, and socially consequential.

Given the substantial uncertainty surrounding artificial intelligence, it is imperative to examine the mechanisms through which trust structures an individual’s perception of AI. Specifically, trust may shape how people evaluate the potential benefits and risks of AI technologies, and these judgments, in turn, are likely to play a decisive role in shaping acceptance of AI.

### 1.1. Differentiated Roles of Trust in Shaping Perceptions

A nuanced understanding of how trust shapes public responses to AI requires distinguishing among multiple layers of trust-related psychology. Prior research has consistently conceptualized trust as a multi-stage process involving at least three analytically distinct components: propensity to trust, referring to a generalized disposition to view a category of actors or technologies as trustworthy; perceived trustworthiness, referring to evaluative judgments toward a specific target; and reliance decisions, referring to the behavioral willingness to accept vulnerability in concrete situations (Mayer et al., 1995; McKnight et al., 2002; Thielmann & Hilbig, 2015). These layers capture different stages through which trust operates, ranging from abstract orientations to concrete behavioral responses.

Existing research on trust in automation has primarily concentrated on the latter two, more downstream stages. This literature examines how trust in specific automated systems develops through interaction and how such trust guides appropriate reliance, misuse, or disuse in operational contexts (Hoff & Bashir, 2015). While these studies provide valuable insights into experience-based trust calibration, they offer more limited guidance for understanding trust formation at more generalized, upstream levels—particularly in contexts where direct experience with specific AI systems is limited.

This limitation is especially salient in the current stage of AI diffusion. Despite a growing research emphasis on domain-specific AI applications, empirical evidence suggests that most citizens remain unfamiliar with many AI technologies (Tang et al., 2025). As a result, public attitudes toward AI are often formed at a generalized or symbolic level rather than grounded in sustained interaction with particular systems. Under such early-stage conditions, individuals are unlikely to rely on system-specific evaluations of trustworthiness; instead, their perceptions and acceptance of AI are more likely shaped by broad, upstream trust orientations toward automated technologies as a class.

Within the layered conceptualization of trust outlined above, this upstream orientation corresponds to propensity to trust automated technologies (PTT-A). PTT-A captures individuals’ generalized predisposition to engage with automated systems, independent of direct experience with any specific application (McKnight & Chervany, 2001; Scholz et al., 2024). Prior research indicates that when experiential information is limited, such generalized trust dispositions play a particularly influential role in shaping attitudes toward emerging technologies (McKnight et al., 2002). Accordingly, focusing on PTT-A provides a theoretically coherent and contextually grounded basis for examining how public trust influences perceptions and acceptance of AI.

PTT-A comprises four theoretically distinct dimensions: trusting stance, competence, benevolence, and integrity. Each captures different aspects of how individuals approach and evaluate automated technologies under conditions of uncertainty. Trusting stance reflects a general orientation toward engagement with automated technologies under uncertainty. By contrast, competence, benevolence, and integrity constitute belief-based trust, capturing generalized expectations about whether automated systems are capable, well-intentioned, and norm-consistent.

To understand how different dimensions of trust shape perceptions of AI, trust must be situated within the cognitive processes by which individuals evaluate communicated information under uncertainty. Epistemic vigilance theory provides such a framework, starting from the premise that communication is inherently risky, as information sources may be mistaken, biased, or strategically misleading, necessitating a regulatory system that continuously monitors epistemic reliability (Mercier, 2020; Sperber et al., 2010). Importantly, epistemic vigilance does not function as a post hoc check that follows information acceptance. Rather, it represents a default, ongoing orientation that governs how individuals remain open to potentially relevant information while simultaneously scrutinizing its credibility. Within this framework, openness reflects a provisional willingness to engage with information rather than uncritical acceptance, whereas vigilance operates throughout the interpretive process by evaluating source reliability, motivational cues, and coherence with existing beliefs (Ding & Ge, 2023). From this perspective, trust is inherently vigilant rather than naive: trust and scrutiny are mutually constitutive processes that jointly regulate belief formation under uncertainty.

Building on this cognitively grounded account, trusting stance plays a foundational role by shaping individuals’ default orientation toward engaging with AI-related information under uncertainty. Within the epistemic vigilance system, trusting stance operates not as a guarantee of acceptance but as an openness bias: it influences whether individuals are willing to attend to, consider, and cognitively engage with AI-related cues when information is incomplete. Individuals high in trusting stance are therefore more inclined to interact with novel, uncertain, or opaque technological agents, exposing them to a broader array of informational signals. This expanded engagement increases the likelihood of recognizing potential advantages—such as convenience, efficiency, or accuracy—while simultaneously heightening awareness of possible downsides. As Ding and Ge (2023) note, greater openness tends to amplify, rather than attenuate, attention to both supportive and contradictory signals. Accordingly, trusting stance may elevate both perceived benefits and perceived risks, reflecting intensified epistemic engagement rather than naive acceptance.

In contrast to a general trusting stance, belief-based trust dimensions shape perceptions primarily through source-related epistemic vigilance—that is, the cognitive processes through which individuals assess whether an information provider is capable, well-intentioned, and norm-compliant (Afroogh et al., 2024; Bedué & Fritzsche, 2022). Among these dimensions, competence captures users’ expectations of what an AI system can do, including its technical capabilities, task expertise, and performance effectiveness (Afroogh et al., 2024; Gilad et al., 2021). When an AI system is perceived as highly competent, users are more likely to expect accurate, efficient, and reliable task performance. These performance-related inferences strengthen expectations of successful outcomes and functional gains, thereby increasing perceived benefits of AI use (Afroogh et al., 2024; Gieselmann & Sassenberg, 2023). At the same time, competence beliefs may also heighten sensitivity to potential downsides. Highly capable systems are often seen as having broader influence, greater autonomy, or wider systemic impact, which can make concerns about loss of human control, unintended large-scale consequences, or privacy risks more salient (M. Li & Bitterly, 2024; Liang & Xue, 2009). Thus, competence belief may increase the salience of both positive and negative future outcomes. Consequently, while competence is likely to be positively associated with perceived benefits, its relationship with perceived risks is theoretically indeterminate and contingent on contextual interpretation of capability.

Whereas competence primarily informs expectations about what an AI system is capable of, benevolence and integrity beliefs shape expectations about what the system will do (Scholz et al., 2024). Benevolence belief reflects users’ inferences regarding an AI system’s intentions—specifically, whether it is motivated to act in the user’s best interests (Erdmann, 2022; Gilad et al., 2021). Within epistemic vigilance, such intention-based beliefs play a central role in source evaluation under uncertainty. When an AI system is perceived as benevolent, users are more inclined to anticipate supportive, user-centered, and cooperative outcomes while holding fewer negative expectations, as concerns about hidden motives, adversarial manipulation, or exploitative intent are attenuated (Afroogh et al., 2024; Bedué & Fritzsche, 2022; Erdmann, 2022). Thus, benevolence belief is likely associated with higher perceived benefits and lower perceived risks.

Integrity belief reflects users’ beliefs that an AI system adheres to accepted principles and normative standards, such as honesty, transparency, and fairness (Mehrotra et al., 2024). As a normative belief, integrity shapes expectations that the system will behave in rule-consistent, accountable, and ethically constrained ways. By fostering confidence in predictable and norm-compliant system behavior, integrity belief enhances perceived benefits by strengthening perceptions of legitimacy, reliability, and social acceptability (Mehrotra et al., 2024). However, because many AI-related risks stem from systemic uncertainty, unintended consequences, or scale-related impacts rather than from intentional misconduct or rule violations, integrity beliefs may not directly resolve broader risk concerns. Hence, while integrity belief may be positively associated with perceived benefits, its association with perceived risks remains context-dependent rather than uniformly risk-dampening.

Taken together, these mechanisms illustrate how the four dimensions of propensity to trust in automation reconfigure epistemic vigilance in distinct ways. Trusting stance primarily regulates openness to information, increasing cognitive engagement with AI-related cues, whereas competence, benevolence, and integrity beliefs selectively shape source- and norm-related vigilance during information evaluation. As a result, trust does not uniformly suppress uncertainty; instead, different trust dimensions channel epistemic vigilance in different directions, producing differentiated patterns of perceived benefits and perceived risks that ultimately shape AI acceptance.

### 1.2. The Role of Perceived Benefits and Risks in AI Acceptance

For evaluative judgments that decisively shape AI acceptance, perceived benefits and perceived risks constitute the two primary appraisal pathways through which individuals form acceptance decisions. In the AI context, perceived benefits typically include anticipated efficiency gains, improved service quality, enhanced convenience, and broader societal value (Brauner et al., 2025; Kouloukoui et al., 2025). In contrast, perceived risks encompass concerns about privacy violations, data misuse, algorithmic bias, opacity, loss of control, and system failures (Al-kfairy et al., 2024). Early research on risk perception conceptualized benefits and risks as a compensatory trade-off, assuming a negative correlation between the two, while technologies perceived as highly beneficial were presumed to be less risky, and vice versa (Alhakami & Slovic, 1994). However, as AI technologies have become increasingly complex and deeply embedded in social systems, this unidimensional assumption has been challenged. While some studies continue to observe a negative association between perceived benefits and perceived risks in AI contexts (Brauner et al., 2025), a growing body of empirical evidence indicates that individuals frequently perceive AI as simultaneously high in both potential benefits and potential risks, reflecting a condition of risk–benefit co-salience (Gerlich, 2023; Schlegel & Uenal, 2021; Schwesig et al., 2023). These findings suggest that attitudes toward AI are often structurally ambivalent rather than unidimensional.

Traditional rational decision-making frameworks are used to model perceived benefits and risks as independent, compensatory predictors of technology acceptance: greater perceived benefits promote acceptance intentions (Ajzen & Fishbein, 2005), whereas greater perceived risks suppress them (Kim et al., 2008; Peter & Tarpey, 1975). These models implicitly assume that individuals can form stable, internally consistent evaluations of risks and benefits, and that risk perception exerts linear, monotonic effects on behavioral responses. However, accumulating evidence demonstrates that such assumptions often fail to hold in AI contexts characterized by uncertainty, opacity, and far-reaching societal implications (Goh et al., 2024).

To address this complexity, dual-processing models provide a more nuanced perspective on AI acceptance. These models propose that individuals rely on two interacting systems when evaluating risky technologies: an analytical, deliberative system that processes probabilistic and outcome-based information, and an experiential, intuitive system that operates rapidly and is closely tied to affective responses (Bellini-Leite, 2022). Acceptance decisions, therefore, emerge from the dynamic interplay between these systems rather than from purely analytical cost–benefit calculations. Within this framework, prior research further shows that different modes of risk representation can yield markedly different behavioral responses. For example, fuzzy-trace theory distinguishes between verbatim representations, which emphasize precise probabilities and outcomes, and gist-based representations, which capture the essential meaning or overall impression of a risk situation. Whereas verbatim, trade-off-oriented processing may lead individuals to accept risky options when perceived benefits outweigh low-probability harms, gist-based processing is more likely to produce categorical judgments that favor protective or avoidance-oriented behaviors (Mills et al., 2008). Together, this body of work underscores that risk evaluation is not exclusively analytical in nature.

These insights from the risk-as-feelings approach place affective responses at the center of risk-related decision-making (Loewenstein et al., 2001). From this perspective, individuals’ reactions to AI-related risks are not always driven by deliberate assessments of probabilities and consequences. Instead, emotions such as fear, anxiety, or unease may be triggered directly by AI risk contexts and can guide behavior independently of, or even before, analytical evaluation. When affective and cognitive appraisals diverge, emotional responses may override deliberate appraisals—a dynamic that is particularly salient in AI contexts where perceived benefits and perceived risks are simultaneously high. Importantly, perceived risk does not inevitably translate into sustained risk-avoidant behavior once protective measures are perceived to be in place. Risk compensation theory suggests that when individuals believe their environment has become safer due to technological safeguards, regulation, or institutional oversight, they may adjust their behavior in a more risk-tolerant direction to maintain a preferred level of risk (Levym & Miller, 2000). Applied to AI governance, this perspective offers a critical extension. In contexts where AI regulation is predominantly government-led (Al-Maamari, 2025) and public trust in authorities is relatively high (Ding & Ge, 2024), individuals may perceive strong external control and institutional protection. Under such conditions, awareness of AI-related risks may coexist with reduced vigilance and increased reliance on AI systems. Consequently, perceived risk may not lead to lower acceptance or more cautious use, as compensatory risk-taking may offset initial concerns. Thus, perceived benefits are consistently and positively associated with AI acceptance, whereas the effect of perceived risks is theoretically ambiguous and context-dependent.

### 1.3. The Present Study

Building on prior theoretical and empirical work, the present study pursues two interrelated research aims. First, it examines how distinct dimensions of trust influence AI acceptance by differentially affecting perceived benefits and risks, and conceptualizes epistemic vigilance as a cognitive mechanism underpinning vigilant forms of trust. Second, it investigates the functional role of perceived risk in AI acceptance, assessing whether risk perception invariably constrains adoption or whether it may coexist with and potentially accompany acceptance under conditions of heightened engagement.

To address these objectives, we draw data from a large-scale, nationally representative household survey (i.e., the 2024–2025 Chinese Social Mentality Survey; CSMS) in China—a society experiencing rapid deployment of AI technologies across finance, healthcare, transportation, and governance (Roberts et al., 2021). The CSMS employed a stratified probability sampling design and conducted in-person household interviews across 31 provinces, thereby mitigating potential digital divide biases commonly associated with online surveys (Fritsch et al., 2022). The survey yielded 10,294 valid responses. Respondents were asked to evaluate eleven representative AI applications—including generative AI, medical robots, and autonomous vehicles—as well as their general attitudes toward AI, encompassing perceived benefits, perceived risks, and multidimensional trust.

**Figure 1:**
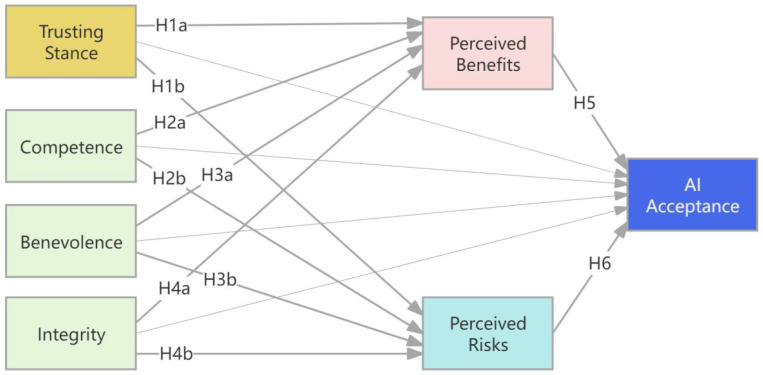
The Psychological Framework of Public Acceptance of AI. Note. Hypotheses 1–6 specify the direct pathways among constructs. Hypotheses 7–8 propose that the effects of trust dimensions on AI acceptance are mediated through perceived benefits and perceived risks, corresponding to the indirect pathways illustrated in the model.

Based on the preceding theoretical arguments, perceived benefits and perceived risks constitute dual appraisal pathways through which PTT-A shapes AI acceptance (see Figure 1). It was first hypothesized that respondents’ trust (including trusting stance, competence belief, benevolence belief, and integrity belief) would all be positively associated with their perceived benefits of AI. Second, it was expected that the relationships between respondents’ trust dimensions and perceived risk would be mixed. While we anticipated that benevolence and integrity beliefs would be negatively correlated with perceived risks, our investigation into the effect of respondents’ trusting stance and competence beliefs on perceived risks remains exploratory. Similarly, while perceived benefits were expected to be positively associated with AI acceptance, the association between perceived risks and AI acceptance was undetermined. Finally, we hypothesized that individuals’ trust would be an antecedent to their acceptance of AI, which operates through the parallel mediators of perceived benefits and risks. The hypotheses are summarized as follows:

**H1a.**  *Trusting stance is positively associated with perceived benefits.*

**H1b.**  *Trusting stance is positively associated with perceived risks.*

**H2a.**  *Competence belief is positively associated with perceived benefits.*

**H2b.**  *The association between competence belief and perceived risks is indeterminate.*

**H3a.**  *Benevolence belief is positively associated with perceived benefits.*

**H3b.**  *Benevolence belief is negatively associated with perceived risks.*

**H4a.**  *Integrity belief is positively associated with perceived benefits.*

**H4b.**  *Integrity belief is negatively associated with perceived risks.*

**H5.**  *Perceived benefits are positively associated with AI acceptance.*

**H6.**  *The association between perceived risks and AI acceptance is ambiguous.*

**H7.**  *Perceived benefits are associated with the relationship between trust dimensions and AI acceptance.*

**H8.**  *Perceived risks are associated with the relationship between trust dimensions and AI acceptance.*

## 2. Methods

### 2.1. Sample and Data

The data for this study were drawn from the 2024–2025 Chinese Social Mentality Survey (CSMS), a nationwide research project led by the Institute of Sociology at the Chinese Academy of Social Sciences. Data collection took place from October 2024 to February 2025, using a stratified sampling design with probability proportional to size (PPS) sampling applied within strata. A total of 145 counties (districts) and 314 urban and rural communities were selected across 31 provinces, autonomous regions, and municipalities in China. Face-to-face household interviews were conducted with residents aged 18–70 years who had resided in their local community for at least six consecutive months. In total, 11,163 individuals were surveyed. After data quality checks and exclusion of 869 invalid responses, a final sample of 10,294 valid responses was obtained.

Because our primary aim was to estimate overall AI acceptance, we excluded respondents who selected the “do not know” option on key items (e.g., the acceptance measures), resulting in a final analytical sample of 6617 individuals in the main analysis. Among the 6617 respondents included (*M*(age) = 41.49 years, *SD* = 12.91), 54.1% were female, and 45.9% were male. Regarding residential status, 4096 respondents (61.9%) lived in urban areas, and 2521 (38.1%) resided in rural areas. Most participants reported holding at least a high school degree (89.5%) at the time of data collection.

### 2.2. Variables and Measurements

This study examined AI acceptance, emphasizing the role of public trust in shaping perceptions of AI benefits and risks, as well as acceptance itself. The key variables and their measurement approaches are described below.

#### 2.2.1. Trust in AI

Trust in AI was measured using the Propensity to Trust in Automated Technology (PTT-A) scale developed by Scholz et al. (2024). The original authors provide both a full 14-item version and a psychometrically validated six-item short form. We employed the short form because the present study was embedded in a large-scale national survey, where questionnaire length is known to substantially affect data quality, response rates, and satisficing behavior. Importantly, the short form was developed and validated by the scale authors and retains the original four theoretically derived trust facets—trusting stance, competence, benevolence, and integrity—using the most representative indicators.

In the six-item short form, trusting stance is assessed with two items, which demonstrated acceptable internal consistency in our data (*r* = 0.70); the mean of these items was therefore used as the composite score for this facet. Benevolence and integrity are each represented by a single, conceptually clear item. Competence is represented by two items: one positively worded competence item and one reverse-coded item reflecting wariness. In the present study, however, these two competence items showed very low inter-item correlation (*r* = 0.13). This pattern is consistent with prior research indicating that reverse-coded items often introduce methodological noise and attenuate internal consistency, particularly at short scales (Podsakoff et al., 2003; Schmitt & Stults, 1985; van Sonderen et al., 2013). Moreover, the wariness item appeared to diverge conceptually from a narrow definition of system competence and to capture a distinct evaluative process. To preserve conceptual clarity and minimize method effects in a brief national survey context, competence was therefore operationalized using only the conceptually precise item (“Automated technological systems are competent”).

This measurement decision is also consistent with prior methodological research on the use of single-item measures. Although multi-item scales remain the gold standard for latent constructs, single-item measures are increasingly recognized as acceptable under certain conditions. Allen et al. (2022) argue that single-item measures may yield valid and reliable data when constructs are narrow, semantically unambiguous, and easily understood by respondents—conditions that apply to concrete beliefs about the competence, benevolence, and integrity of automated systems. Empirical evidence supports this position. For example, Cheung and Lucas (2014) showed that single-item life satisfaction measures exhibit strong criterion and construct validity comparable to multi-item scales in large, nationally representative samples. Methodological reviews further suggest that when respondent burden is a concern, as in large-scale surveys, single-item measures—particularly when drawn from previously validated multi-item scales—can offer a reasonable trade-off between brevity and measurement quality (Spörrle & Bekk, 2014). In line with these considerations, the three belief-based facets (competence, benevolence, and integrity) were modeled as single-item observed variables rather than latent constructs in the present study.

#### 2.2.2. Perceived Benefits and Risks of AI

Perceived benefits refer to the positive outcomes that individuals expect from the integration of AI into their daily lives, including increased convenience, enhanced work efficiency, and cost reduction. Participants’ perceptions of these benefits were assessed using a 6-item scale adapted from Gerlich (2023), which measures improvements in convenience, work efficiency, and cost reduction due to AI. Each item was rated on a 7-point Likert scale (1 = strongly disagree, 7 = strongly agree), with an additional option of 8 indicating insufficient understanding to provide a rating. The mean score of all six items was computed to form a composite measure (Cronbach’s α = 0.79).

Perceived risks refer to the negative consequences respondents associate with AI, such as concerns regarding job displacement, privacy violations, algorithmic bias, and broader ethical challenges (Gerlich, 2023). Drawing on prior research that has identified these themes as central to public concerns about AI, an eight-item scale was adapted to comprehensively capture perceptions of AI-related concerns. The scale included eight items, each rated on a 7-point Likert scale (1 = strongly disagree, 7 = strongly agree), with the composite score calculated as the average of all eight items (Cronbach’s α = 0.82).

#### 2.2.3. AI Acceptance

Public acceptance of AI was assessed through respondents’ willingness to adopt eleven representative AI applications that reflect the breadth of contemporary technological integration. These applications covered both general-purpose tools—such as generative AI and chatbots—and domain-specific systems, including medical robots, autonomous vehicles, financial algorithms, and AI-assisted public services. For each application, participants rated its acceptability on a 7-point Likert scale (1 = completely unacceptable, 7 = completely acceptable). An additional option, “8 = Do not know,” was also provided.^1^

Across the full sample (*N* = 10,294), acceptance levels varied considerably across applications, revealing meaningful nuances in public perceptions of AI. As shown in Table 1, mean acceptance scores ranged from 3.96 to 5.66 (*SD* = 1.32–1.59). The highest acceptance was observed for household robots (*M* = 5.66, *SD* = 1.32), indicating strong receptivity toward AI systems that provide tangible, everyday utility. In contrast, acceptance was markedly lower for virtual companions (*M* = 3.96, *SD* = 1.55) and virtual anchors or livestream hosts (*M* = 4.00, *SD* = 1.59), suggesting greater public hesitation toward socially embedded or anthropomorphic AI applications that challenge human–machine boundaries.

Given this heterogeneity, we computed an aggregate AI acceptance index by averaging participants’ ratings across the eleven items (Cronbach’s α = 0.85). This composite measure provides a reliable and theoretically justified indicator of generalized acceptance of AI, capturing individuals’ overall orientation toward AI technologies rather than attitudes confined to specific domains.

#### 2.2.4. Control Variables

To determine appropriate control variables, a multi-factor ANCOVA was conducted with AI acceptance as the dependent variable, examining demographic predictors such as AI literacy, gender, age, educational attainment, and residential type. AI literacy emerged as the most significant predictor (F(1, 6591) = 1397.66, *p* < 0.001, η^2^ = 0.175) and was thus retained as a covariate in all subsequent analyses due to its strong and consistent associations with other key constructs (*r* = 0.12–0.49). Despite its statistical significance (F(5, 6591) = 18.03, *p* < 0.001, η^2^ = 0.013), educational attainment was excluded primarily to maintain model parsimony, as its effect size was minimal. Other demographic variables, including gender, residential type, and their interactions, were not significant (*p* > 0.05) and were excluded to maintain consistency in model parsimony.

AI literacy, as the key control variable, was measured using a concise 4-item scale developed by B. Wang et al. (2023), with all items rated on a 7-point Likert scale. The variable was calculated as the average of four items (Cronbach’s α = 0.70).

#### 2.2.5. Common Method Variance

Because all variables were self-reported and collected in a single survey session, we conducted Harman’s single-factor test to assess common method variance. An exploratory factor analysis using unrotated principal component extraction showed that the first factor accounted for 21.50% of the total variance, which is far below the recommended threshold of 50%. Therefore, common method variance is unlikely to pose a serious threat to the validity of our results.

#### 2.2.6. Data Analyses

Descriptive analyses were performed to examine the distributional properties of the key constructs, including the four dimensions of trust (trusting stance, competence, benevolence, and integrity), perceived benefits, perceived risks, AI acceptance, and AI literacy. Bivariate Pearson correlations were computed to assess the initial associations among these variables. To test the direct and indirect effects of trust on AI acceptance, a saturated mediation model was estimated, with AI literacy included as a covariate for all endogenous variables. Finally, we conducted robustness checks by estimating the same model separately for the AI applications that respondents reported having heard about the most and the least.

## 3. Results

### 3.1. Descriptive Statistics

As shown in Table 2, respondents exhibited a generally positive attitude towards AI, with the AI acceptance score being moderately high (*M* = 4.58). Additionally, respondents reported moderately high scores on the perceived benefits (*M* = 4.97) and perceived risks (*M* = 4.50) of AI, indicating that participants simultaneously recognized the advantages of AI technologies while maintaining a cautious awareness of potential risks. Among the four dimensions of trust, the competence (*M* = 4.69) was the highest, followed by the integrity (*M* = 4.54), the trusting stance (*M* = 4.30), and the benevolence (*M* = 4.12).

### 3.2. Associations Between Main Study Variables

Pearson’s correlation analyses were conducted to explore the relationships among the key variables (Table 3). AI acceptance showed positive correlations with all four trust dimensions (trusting stance, competence, benevolence, and integrity; *p*(s) < 0.001). Each trust dimension was also significantly associated with perceived benefits, with particularly strong correlations observed for integrity (*r* = 0.41) and competence (*r* = 0.40). All dimensions of trust were positively significantly associated with perceived risks, except for benevolence. As expected, perceived benefits were strongly and positively correlated with AI acceptance (*r* = 0.49). However, contrary to expectation, perceived risks also showed a significant positive correlation with AI acceptance (*r* = 0.11).

### 3.3. Regression Analysis

To examine how trust-related antecedents shape users’ cognitive evaluations of AI, we first regressed perceived benefits and perceived risks on the four trust dimensions—trusting stance, competence, benevolence, and integrity—while controlling for literacy. The model predicting perceived benefits demonstrated substantial explanatory power (R^2^ = 0.3446, *p* < 0.001). All four trust dimensions positively predicted perceived benefits: trusting stance (*β* = 0.0234, *p* = 0.0155 < 0.05), competence (*β* = 0.1336, *p* < 0.001), benevolence (*β* = 0.0280, *p* = 0.0006 < 0.001), and integrity (*β* = 0.1557, *p* < 0.001). The perceived risk model showed lower but significant explanatory power (R^2^ = 0.0900, *p* < 0.001). Benevolence strongly reduced perceived risks (*β* = −0.1979, *p* < 0.001), whereas trusting stance increased them (*β* = 0.0664, *p* < 0.001). Competence (*β* = 0.0174, *p* = 0.1216) and integrity (*β* = −0.0187, *p* = 0.1136) did not significantly predict perceived risks. Literacy significantly predicted both benefits (*β* = 0.3961, *p* < 0.001) and risks (*β* = 0.1691, *p* < 0.001), indicating that users with higher AI literacy tend to perceive both advantages and potential risks more clearly.

Next, we regressed AI acceptance on the four trust antecedents together with the two mediators. The model showed substantial explanatory power (R^2^ = 0.3182, *p* < 0.001). All four trust antecedents exerted significant direct effects on AI acceptance, controlling for the other variables: trusting stance (*β* = 0.0304, *p* = 0.0028 < 0.05), competence (*β* = 0.0484, *p* < 0.001), benevolence (*β* = 0.0244, *p* = 0.0064 < 0.05), and integrity (*β* = 0.0557, *p* < 0.001). Perceived benefits were the strongest positive predictor (*β* = 0.3004, *p* < 0.001), while perceived risks also demonstrated a significant positive effect (*β* = 0.0553, *p* < 0.001), suggesting that higher awareness of AI’s risks may coexist with, rather than suppress, acceptance in this sample. Literacy again showed a strong positive influence (*β* = 0.2773, *p* < 0.001).

To further assess the mediating mechanisms, we conducted four separate mediation analyses using PROCESS Model 4 (Hayes, 2022), each treating one trust antecedent as the focal predictor, with perceived benefits and perceived risks as parallel mediators. The remaining three trust antecedents and literacy were included as covariates. Indirect effects were tested using 5000 bootstrap samples and percentile confidence intervals.

Trusting stance exhibited a significant total indirect effect on AI acceptance (effect = 0.0107, 95% CI [0.0042, 0.0171]). Both mediators contributed to this effect: perceived benefits (effect = 0.0070, 95% CI [0.0008, 0.0132]) and perceived risks (effect = 0.0037, 95% CI [0.0018, 0.0058]). The direct effect remained significant (*β* = 0.0304, *p* = 0.0028), indicating partial mediation.

Competence showed a strong and significant total indirect effect (effect = 0.0411, 95% CI [0.0335, 0.0492]) that is almost entirely driven by perceived benefits (effect = 0.0402, 95% CI [0.0328, 0.0478]). The indirect effect via perceived risks was not significant. A significant direct effect also emerged (*β* = 0.0484, *p* < 0.001).

Benevolence displayed a dual-path pattern with two indirect effects in opposite directions. The benefit pathway produced a significant positive indirect effect (effect = 0.0084, 95% CI [0.0034, 0.0137]), whereas the risk pathway produced a significant negative indirect effect (effect = −0.0109, 95% CI [−0.0156, −0.0062]). These opposing pathways resulted in a nonsignificant total indirect effect (effect = −0.0025, 95% CI [−0.0095, 0.0044]). The direct effect remained significant (*β* = 0.0244, *p* = 0.0064).

Integrity demonstrated a significant total indirect effect (effect = 0.0458, 95% CI [0.0377, 0.0539]), primarily via perceived benefits (effect = 0.0468, 95% CI [0.0387, 0.0547]). The risk pathway was not significant. The direct effect was also significant (*β* = 0.0557, *p* < 0.001).

Taken together (see Table 4), the mediation analyses reveal that perceived benefits serve as the most consistent and powerful mediator across all four trust antecedents. Perceived risks play a more nuanced and inconsistent role, producing a positive mediating effect for trusting stance, a negative mediating effect for benevolence, and no significant effects for competence or integrity. Integrity and competence exert the strongest total effects (direct + indirect) on AI acceptance. Benevolence exhibits a theoretically meaningful “dual-path” structure, with benefit and risk pathways offsetting each other. Literacy remains a robust control variable predicting benefits, risks, and AI acceptance in every model. These results lend strong empirical support to the overall model in which trust dimensions shape AI acceptance primarily through the cognitive evaluation of AI’s benefits and, to a lesser extent, risks.

### 3.4. Robustness Check Across Widely Heard-Of and Least Heard-Of Products

Analyses conducted on AI products that are widely heard of in society and those that are least heard of yielded a highly consistent pattern. The following table (Table 5) presents the awareness levels of different AI technologies.

Across both product types, perceived benefits remained the strongest and most reliable predictor of AI acceptance (*β* = 0.3259 and *β* = 0.3919, respectively). This indicates that adoption decisions are fundamentally driven by expectations of positive outcomes rather than by the broader societal prominence of a specific AI application.

Trust dimensions also exerted parallel effects on benefit perceptions across product types. Trusting stance, competence, benevolence, and integrity each significantly increased perceived benefits. In turn, perceived benefits provided a robust mediating pathway through which trust fostered acceptance. This replication across product types highlights benefit appraisal as the core psychological mechanism linking trust to acceptance. Details are reported in Table 6.

The pattern for perceived risks was similarly stable but more nuanced. Benevolence consistently reduced perceived risks, whereas trusting stance and competence increased them, with integrity showing no significant influence in either model. However, the behavioral relevance of perceived risk differed between product types. For widely heard-of products, risk perceptions unexpectedly predicted higher acceptance, producing both positive and negative indirect effects depending on the specific trust dimension. For the least heard-of products, perceived risk did not significantly predict acceptance, and the indirect path through risk remained null.

Taken together, the robustness analyses confirm that the pathway linking trust to perceived benefits and, in turn, to acceptance, remains stable across contexts and constitutes the dominant mechanism across AI product types with varying levels of societal recognition. The relationship between trust and perceived risk is also directionally consistent, although its influence on acceptance varies by product type. Collectively, these findings provide strong evidence for the theoretical stability of the vigilant trust model across heterogeneous AI product categories. Additional details are presented in Table 6.

### 3.5. Hypothesis Testing Results

Based on the main analysis and robustness checks, H1a, H1b, H2a, H3a, H3b, H4a, H5, and H7 were fully supported. H2b and H6 received partial support: competence belief was associated with perceived risks only under certain conditions, and the relationship between perceived risks and AI acceptance varied across product types. In contrast, H4b and H8 were not supported, as integrity belief did not significantly predict perceived risks, nor did perceived risks mediate the relationship between trust dimensions and AI acceptance in either the main analysis or the robustness checks.

## 4. Discussion

This study examined how public trust in AI shapes acceptance through differentiated perceptions of benefits and risks. Building on epistemic vigilance theory (Mercier, 2020; Sperber et al., 2010), we proposed a vigilant trust framework in which trust is conceptualized not as a uniform risk-dampening disposition but as a multidimensional orientation that structures how individuals engage with and evaluate AI-related information under uncertainty. Specifically, we distinguished between trusting stance, which reflects a generalized openness toward automated systems (McKnight et al., 2002; Scholz et al., 2024), and belief-based trust dimensions—competence, benevolence, and integrity—which capture generalized expectations about system capability, intentions, and norm compliance. Within this framework, perceived benefits and perceived risks are treated as parallel cognitive outcomes of epistemically vigilant information processing rather than as simple trade-offs. The empirical results provide consistent support for this conceptualization.

### 4.1. Vigilant Trust and Divergent Pathways

The findings show that the four dimensions of propensity to trust automated technologies exert differentiated effects on perceived benefits and perceived risks, consistent with their theoretically distinct roles in epistemic vigilance.

Trusting stance was positively associated with both perceived benefits and perceived risks across the main analysis and robustness checks. This pattern suggests that trusting stance does not attenuate concern but instead increases cognitive engagement with AI-related information. Such openness-oriented engagement is central to epistemic vigilance, which posits that admitting information into the cognitive system enables both opportunity recognition and critical scrutiny (Mercier, 2020; Sperber et al., 2010). Individuals with a stronger trusting stance may therefore be more likely to recognize AI’s potential advantages while simultaneously remaining attentive to uncertainty and possible negative consequences.

Competence consistently increased perceived benefits, but did not reliably reduce perceived risks. In one robustness model, higher competence belief was even associated with higher perceived risks for certain applications. This conditional pattern suggests that perceiving AI as highly capable may heighten awareness of its scale, autonomy, or systemic impact, thereby rendering certain risks more salient (Y. Li et al., 2025; Liang & Xue, 2009). Importantly, this association was not uniform across models, underscoring the context-dependent nature of competence-related risk sensitivity.

Benevolence exhibited a more consistent risk-dampening role. Expectations that AI systems act in users’ best interests were associated with higher perceived benefits and lower perceived risks, aligning with prior research showing that goodwill reduces uncertainty and mitigates concerns about opportunistic or harmful intentions (Bedué & Fritzsche, 2022; Erdmann, 2022).

Integrity belief showed a more selective pattern. Expectations that AI systems adhere to principles such as fairness, transparency, and accountability were consistently associated with higher perceived benefits. However, integrity did not exhibit a significant association with perceived risks in the main analysis or robustness checks. This pattern indicates that while normative assurances may strengthen positive expectations about AI, they do not necessarily resolve concerns related to uncertainty, system failures, or unintended consequences. In this sense, integrity appears to contribute to benefit recognition without functioning as a direct risk-attenuating mechanism, further underscoring that trust dimensions do not uniformly suppress vigilance but shape benefit–risk evaluations in differentiated ways.

### 4.2. Reinterpreting the Risk–Acceptance Relationship

Across all models, perceived benefits emerged as the strongest and most stable predictor of AI acceptance. Individuals were consistently more willing to accept AI when they anticipated efficiency gains, improved service quality, or broader societal value, a pattern that aligns with expectancy–value traditions and prior technology acceptance research (Ajzen & Fishbein, 2005; Bronfman & López-Vázquez, 2011).

In contrast, perceived risks did not function as a consistent deterrent. In the main model and one robustness specification, perceived risks were even positively associated with AI acceptance, while in the least familiar application, this relationship became non-significant. Rather than viewing the positive or non-deterrent association as anomalous, we outline three theoretically grounded explanations that are compatible with the vigilant trust framework.

First, a reflective engagement explanation suggests that higher perceived risk may signal deeper cognitive involvement rather than aversion. Individuals who actively seek information and scrutinize AI systems are likely to recognize both benefits and risks, reflecting an epistemically vigilant mode of reasoning in which openness to information heightens critical evaluation (Ding & Ge, 2023; Mercier, 2020; Sperber et al., 2010). Under this interpretation, risk awareness accompanies acceptance because both emerge from sustained engagement rather than avoidance.

Second, a benefit-dominance explanation emphasizes the asymmetric influence of benefits and risks on acceptance decisions. When both evaluations are salient, behavior tends to be more strongly driven by perceived benefits, particularly in innovation-oriented contexts where anticipated gains are substantial (Alhakami & Slovic, 1994). In such cases, individuals may acknowledge considerable risks yet still accept AI because expected benefits outweigh perceived downsides.

Third, a contextual normalization explanation highlights the broader sociotechnical environment in which AI is embedded. In contexts characterized by rapid deployment, institutional endorsement, and strong narratives of technological progress, risks may be perceived as an inherent and unavoidable feature of innovation rather than a prohibitive signal. Similar dynamics have been documented in the “risk perception paradox” literature, where high risk awareness does not consistently translate into avoidance behavior (Wachinger et al., 2013).

These explanations are conceptually distinct and carry different implications for governance, communication, and public engagement. Distinguishing among them requires future research designs that move beyond cross-sectional associations, including longitudinal analyses, experimental manipulations of risk framing, and qualitative investigations into how individuals interpret and negotiate AI-related uncertainty over time.

### 4.3. Implications and Limitations

Overall, the findings demonstrate that trust in AI does not operate as a uniform risk-dampening mechanism. Instead, trust constitutes a differentiated evaluative structure in which openness, belief-based expectations, benefit recognition, and risk vigilance are jointly configured. By showing that trust can heighten vigilance and that risk awareness can coexist with acceptance, this study advances a vigilant trust framework that challenges linear assumptions in technology acceptance research and offers a more realistic account of how individuals reason about emerging sociotechnical systems under uncertainty.

From a theoretical perspective, this framework extends existing trust-based acceptance models by reconceptualizing trust not as the opposite of caution, but as a condition under which critical engagement becomes possible. Importantly, this contribution is twofold: it demonstrates that trust and vigilance can coexist as parallel cognitive processes, and it shows that this coexistence is structured differently by distinct trust dimensions. In particular, trustor-based trusting stance and system-based belief-oriented trust (competence, benevolence, and integrity) play differentiated roles in shaping how individuals attend to benefits and risks. Rather than assuming that trust suppresses uncertainty, the vigilant trust framework highlights how different trust dimensions organize attention to both opportunities and potential harms. This perspective helps reconcile previously inconsistent findings regarding the roles of trust and risk in AI acceptance and provides a cognitively grounded account of why trust and concern may coexist.

From a practical perspective, the findings suggest that efforts to promote public acceptance of AI should not focus solely on alleviating perceived risks. Instead, governance and communication strategies should aim to foster informed and reflective engagement, supporting both benefit recognition and risk awareness. Strengthening public trust through transparency, competence, and benevolence may encourage adoption not by eliminating concern, but by enabling individuals to engage with AI in a more attentive and critically informed manner.

Despite its contributions, this study has several limitations that should be acknowledged and addressed in future research. First, the study relies on a cross-sectional survey design, which limits the ability to draw strong causal inferences regarding the relationships among trust dimensions, perceived benefits, perceived risks, and AI acceptance.

Second, the effect sizes of individual trust dimensions were relatively small. This pattern should be interpreted in light of the upstream and generalized nature of the trust constructs examined, which capture broad orientations toward automated technologies rather than proximal determinants of specific adoption decisions. As a result, their direct effects on acceptance are necessarily modest. Future research could examine more context-specific trust judgments or employ experimental designs to assess whether stronger effects emerge at more proximal levels of decision-making.

Third, while this study advances the concept of vigilant trust, the construct was theoretically inferred through patterned relationships among trust dimensions, perceived benefits, and perceived risks, rather than directly measured using a dedicated psychometric instrument. This modeling strategy is appropriate for an initial theory-testing effort, but it also highlights the need for future research to develop and validate a dedicated vigilant trust scale that explicitly captures the coexistence of openness and scrutiny at the cognitive level.

Finally, the present analysis did not formally test measurement invariance across demographic groups, such as age, gender, education, or levels of AI literacy. As perceptions of AI and trust in automated systems may vary systematically across social groups, future research should examine whether the measurement structure and functional relationships proposed in the vigilant trust model hold consistently across populations.

## 5. Conclusions

This study investigated how multidimensional trust—trusting stance, competence, benevolence, and integrity—shapes public perceptions of AI’s benefits and risks and, in turn, influences acceptance. Using a nationally representative Chinese survey, we found that target-based trust primarily promotes acceptance through enhanced benefit perception, whereas trusting stance amplifies both benefits and risks, supporting the notion of vigilant trust. Importantly, perceived risks did not deter acceptance when benefits were salient. Overall, AI acceptance appears to be driven not by blind optimism or simple trade-offs but by a dual awareness of opportunities and risks, highlighting the need for governance and communication strategies that foster critical, literate, and trust-aware publics.

## Figures and Tables

**Table 1:** Acceptance for each AI application.

Acceptance Statistic	AI Products										
	1	2	3	4	5	6	7	8	9	10	11
*N* (valid)	8113	10,040	9469	9311	9388	9789	9803	8832	8881	9582	9013
Mean	4.58	5.66	4.68	4.42	4.95	4.55	4.98	4	3.96	4.86	4.2
*SD*	1.51	1.32	1.53	1.5	1.46	1.56	1.4	1.59	1.55	1.4	1.51

Notes. 1 = Generative AI (such as ChatGPT and Wenxin Yiyan); 2 = Household robots (such as robot vacuums); 3 = Medical robots; 4 = Robot nannies/friends; 5 = Industrial robots; 6 = Unmanned vehicles (such as self-driving cars); 7 = Smart vehicles (such as smart cars, but with human assistance); 8 = Virtual anchors; 9 = Virtual companions/friends (non-physical); 10 = Smart education products (such as smart tutors and tutoring robots); 11 = Humanoid robots/humanoid robots.

**Table 2:** Descriptive statistics (*N* = 6617).

Variables	Minimum	Maximum	Mean	Standard Deviation
AI Acceptance	1.00	7.00	4.58	0.96
Perceived Benefits	1.17	7.00	4.97	0.93
Perceived Risks	1.00	7.00	4.50	0.88
Trusting Stance	1.00	7.00	4.30	1.19
Competence	1.00	7.00	4.69	1.16
Benevolence	1.00	7.00	4.12	1.20
Integrity	1.00	7.00	4.54	1.13
AI Literacy	1.00	7.00	4.67	0.89

**Table 3:** Associations among main study variables (*N* = 6617).

Variables	1	2	3	4	5	6	7	8
1. AI Acceptance	—							
2. Perceived Benefits of AI	0.488 **	—						
3. Perceived Risks of AI	0.110 **	0.065 **	—					
4. Trusting Stance	0.262 **	0.306 **	0.038 **	—				
5. Competence	0.311 **	0.400 **	0.036 **	0.465 **	—			
6. Benevolence	0.139 **	0.169 **	−0.219 **	0.339 **	0.236 **	—		
7. Integrity	0.317 **	0.411 **	0.027 *	0.506 **	0.554 **	0.205 **	—	
8. AI Literacy	0.456 **	0.490 **	0.160 **	0.242 **	0.283 **	0.118 **	0.284 **	—

Note. ** *p* < 0.01, * *p* < 0.05.

**Table 4:** Direct, indirect, and total effects of trust antecedents on AI acceptance (*N* = 6617).

Predictor	Effect Type	Effect	(Boot) SE	95% CI LL	95% CI UL	Sig.
Trusting stance	Direct	0.0304	0.0102	0.0105	0.0504	Yes
	Indirect (Benefits)	0.007	0.0032	0.0008	0.0132	Yes
	Indirect (Risks)	0.0037	0.001	0.0018	0.0058	Yes
	Total indirect	0.0107	0.0033	0.0042	0.0171	Yes
Competence	Direct	0.0484	0.0107	0.0274	0.0694	Yes
	Indirect (Benefits)	0.0402	0.0039	0.0328	0.0478	Yes
	Indirect (Risks)	0.001	0.0007	−0.0003	0.0024	No
	Total indirect	0.0411	0.004	0.0335	0.0492	Yes
Benevolence	Direct	0.0244	0.0089	0.0069	0.0419	Yes
	Indirect (Benefits)	0.0084	0.0026	0.0034	0.0137	Yes
	Indirect (Risks)	−0.0109	0.0024	−0.0156	−0.0062	Yes
	Total indirect	−0.0025	0.0035	−0.0095	0.0044	No
Integrity	Direct	0.0557	0.0113	0.0336	0.0778	Yes
	Indirect (Benefits)	0.0468	0.0041	0.0387	0.0547	Yes
	Indirect (Risks)	−0.0010	0.0007	−0.0026	0.0002	No
	Total indirect	0.0458	0.0041	0.0377	0.0539	Yes

Table Note. All models controlled for the remaining three trust antecedents and literacy. Indirect effects were estimated using percentile bootstrap confidence intervals with 5000 samples. Confidence intervals that do not include zero indicate statistical significance at the 95% level.

**Table 5:** Awareness of different AI technologies (*N* = 10,294).

Statistic/AI Tech	1	2	3	4	5	6	7	8	9	10	11
Never heard (%)	88.4	37.4	76.5	84.1	72.9	55.1	59.7	84.4	89.7	72.1	79.8
Have heard (%)	11.6	62.6	23.5	15.9	27.1	44.9	40.3	15.6	10.3	27.9	20.2

Notes. The AI product names corresponding to serial numbers 1–11 are consistent with those marked in Table 1.

**Table 6:** Path coefficients of the most familiar (*N* = 9027) and least familiar AI products (*N* = 8139).

	Predictor	Most Familiar Product	Least Familiar Product
Effects of Predictors on Perceived Benefits	Trusting stance	0.0544 (*p* < 0.001)	0.0393 (*p* < 0.001)
	Competence	0.1238 (*p* < 0.001)	0.1326 (*p* < 0.001)
	Benevolence	0.0189 (*p* = 0.0074)	0.0291 (*p* = 0.0001)
	Integrity	0.1527 (*p* < 0.001)	0.1542 (*p* < 0.001)
	AI literacy	0.3462 (*p* < 0.001)	0.3739 (*p* < 0.001)
Indirect Effects via Perceived Benefits	Trusting stance	0.0177 (95% CI [0.0118, 0.0239])	0.0154 (95% CI [0.0080, 0.0230])
	Competence	0.0403 (95% CI [0.0334, 0.0478])	0.0520 (95% CI [0.0425, 0.0624])
	Benevolence	0.0062 (95% CI [0.0015, 0.0108])	0.0114 (95% CI [0.0055, 0.0173])
	Integrity	0.0498 (95% CI [0.0417, 0.0584])	0.0604 (95% CI [0.0506, 0.0704])
Effects of Predictors on Perceived Risk	Trusting stance	0.0335 (*p* = 0.0002)	0.0498 (*p* < 0.001)
	Competence	0.0321 (*p* = 0.0007)	0.0248 (*p* = 0.0131)
	Benevolence	−0.1642 (*p* < 0.001)	−0.1825 (*p* < 0.001)
	Integrity	−0.0140 (n.s.)	−0.0095 (n.s.)
	AI literacy	0.1257 (*p* < 0.001)	0.1342 (*p* < 0.001)
Indirect Effects via Perceived Risk	Trusting stance	0.0040 (95% CI [0.0017, 0.0066])	−0.0016 (95% CI [−0.0037, 0.0003], n.s.)
	Competence	0.0039 (95% CI [0.0016, 0.0065])	−0.0008 (95% CI [−0.0022, 0.0001], n.s.)
	Benevolence	−0.0198 (95% CI [−0.0253, −0.0146])	0.0057 (95% CI [−0.0012, 0.0127], n.s.)
	Integrity	−0.0017 (95% CI [−0.0042, 0.0007], n.s.)	0.0003 (95% CI [−0.0004, 0.0013], n.s.)

Notes. n.s. = not significant.

## Data Availability

The data supporting the findings of this study are available from the corresponding author upon reasonable request.

## Note

1 Although the overall awareness of AI Products was relatively low, most participants were still able to provide a rating on the acceptance scale. This pattern is consistent with survey methodology research, which shows that respondents often distinguish between “having heard of” a product and “being unable to evaluate” it. In many cases, even without prior knowledge, participants can form an impression-based judgment based on the product name, category associations, or general preferences. Therefore, only a small proportion of respondents selected the option “8 = Do not know,” which indicates a complete inability to form any evaluation.
